# Relationship between Atherogenic Dyslipidaemia and Lipid Triad with Different Scales of Overweight and Obesity in 418,343 Spanish Workers

**DOI:** 10.1155/2022/9946255

**Published:** 2022-08-09

**Authors:** Carla Busquets-Cortés, Carlos López, Hernán Paublini, Sebastiana Arroyo Bote, Ángel Arturo López-González, José Ignacio Ramírez-Manent

**Affiliations:** ^1^ADEMA University School, Carrer de Passamaners 11, Palma 07009, Balearic Islands, Spain; ^2^Prevention of Occupational Risks in Health Services, Balearic Islands Health Service, Palma, Spain; ^3^Balearic Islands Health Service, Family Medicine, Calvià, Spain; ^4^Faculty of Medicine, University of the Balearic Islands, Palma, Spain

## Abstract

Obesity induces alterations in lipid biochemistry, evolving toward dyslipidaemia atherogenesis, a critical factor in the development of cardiovascular events. Two relevant forms of lipid abnormalities are atherogenic dyslipidaemia (AD) and lipid triad (LT), which involve alterations in triglyceride levels, HDL-c, and LDL-c. The aim of this study was to assess the linkage of atherogenic AD and LT with different scales of overweight and obesity. We carried out a cross-sectional study including 418,343 Spanish adult workers, recruited from workplace health assessments. Atherogenic dyslipidaemia was defined as triglyceride levels ≥ 150 mg/dL, HDL values < 40 mg/dL in men and <45 mg/dL in women, and normal LDL. Additionally, if LDL levels were >160 mg/dL, LT was considered. Subjects affected by AD and LT in the study exhibited significantly higher mean values than those without AD and LT in all overweight, obesity, and body fat related scales studied. VAI (visceral adiposity index) was the strongest predictor of AD (AUC = 0.934, 95% CI: 0.933 to 0.936) and LT (AUC = 0.926, 95% CI: 0.923 to 0.928). Atherogenic dyslipidaemia and LT positively correlate with different scales of overweight and obesity. Further studies should aim to identify other contributory factors. Our obtained data might be useful in laying the groundwork for future works on AD and LT.

## 1. Introduction

Overweight and obesity are reaching epidemic proportions worldwide [1], since in every single country in the world the incidence of obesity is rising continuously [2]. This abnormal excessive fat accumulation condition is associated with other comorbid conditions that include coronary artery disease, hypertension, type 2 diabetes mellitus, respiratory disorders, and dyslipidaemia [3]. This scenario affects not only developed nations but also lower-middle-income populations, threatening the health systems sustainability. Furthermore, besides extra healthcare burden, which is expected to increase, obesity also entails costs in the form of wasted efficiency as a result of lost workdays, lower productivity at work, higher mortality, and permanent disability conditions [4].

The classification of overweight or obesity according to the body mass index (BMI) has been commonly employed, even though other scales, such as waist circumference (WC), waist to height ratio (WtHR), and University of Navarra Clinic-Body Fat Estimator (CUN-BAE) are emerging to be considered as novel indicators in clinical research for the diagnosis of obesity [5]. Other indicators, such as Equation Córdoba for Estimation of Body Fat (ECORE-BF), relative fat mass (RFM), Palafolls formula, Deurenberg fat mass index, body roundness index (BRI), body shape index (ABSI), visceral adiposity index (VAI) [6], conicity index, normalized weight-adjusted index (NWAI), body surface index (BSI), body surfaces area (BSA), or body fat index (BFI), are used for stratification into the different categories of overweight and obesity, according to gender differences in body composition.

It is widely accepted that obesity induces alterations in lipid biochemistry, evolving toward dyslipidaemia atherogenesis, a critical factor in the development of cardiovascular events [1]. The term “atherogenic dyslipidaemia” (AD) describes elevated levels of triglycerides (TG) and small, dense low-density lipoprotein (LDL-c) and low levels of high-density lipoprotein cholesterol (HDL-c). Additionally, increased levels of large TG-rich very low-density lipoproteins (VLDL), apolipoprotein B, and oxidised low-density lipoprotein and reduced levels of small HDL perform a crucial role in AD. On the other hand, the term “lipid triad” (LT) describes a common form of dyslipidaemia, characterized by three lipid abnormalities: increased plasma TG levels; decreased HDL-c concentrations; and presence of small, dense LDL-c particles. Since this LT generally appears in individuals with cardiovascular disorders, it has been designated as the “atherogenic lipoprotein phenotype” [7].

Several features of obesity and visceral adiposity, such as increased fasting plasma TG, high LDL-c, low HDL-c, elevated fasting plasma glucose (FPG) and insulin levels, and high blood pressure, are associated with augmented cardiovascular risk. There have been described obesity-related novel lipid metabolic risk factors—such as the presence of the small, dense LDL-c phenotype together with postprandial hyperlipidaemia; accumulation of atherogenic remnants; and hepatic overproduction of apoprotein B (apoB)—which are also considered common features of the metabolic syndrome. These lipid abnormalities may be associated with a proinflammatory cytokine gradient, including tumour necrosis factor alpha (TNF-*α*) and interleukin 6 (IL-6) action, which could directly affect the endothelium and promote the process of atherosclerotic plaque formation [2, 8, 9]. The typical dyslipidaemia of obesity consists of increased TG and free fatty acids (FFA); decreased HDL-c with HDL-c dysfunction; and normal or slightly increased LDL-c with increased small, dense LDL-c. The concentrations of plasma apoB are also often increased, partly due to the hepatic overproduction of apoB containing lipoproteins.

Targeting AD is of great interest as it is associated with highly prevalent cardiometabolic disorders in the general population which are commonly accompanied by a high cardiovascular risk (CVR), such as overweight (37%), obesity (17%), diabetes (14%), and metabolic syndrome (30%) [10]. Prevalence of atherogenic dyslipidaemia in Spanish population is relatively high: it is present in 34% of diabetics, 21% of high-risk patients with controlling LDL-c, 10% of hypertensive individuals, and 21–34% of patients with a history of vascular disease (coronary, cerebral, or peripheral arterial) [11].

To the best of our knowledge, no studies on AD and LT have been conducted regarding the relationship of these lipid disbalances with scales of overweight and obesity. Work-related health examinations are a useful opportunity for prevention activities of cardiovascular and metabolic diseases since they permit the identification of the workers at risk in ages in which they usually do not attend sanitary services so frequently. These early findings of risk factors may allow the implementation of preventive measures and actions, especially regarding promotion of healthy habits, preventing these forms of dyslipidaemias in the early stages of life [12].

The aim of this study was to assess the linkage of AD and LT with different scales of overweight and obesity.

## 2. Materials and Methods

### 2.1. Participants in the Study

**Figure 1:**
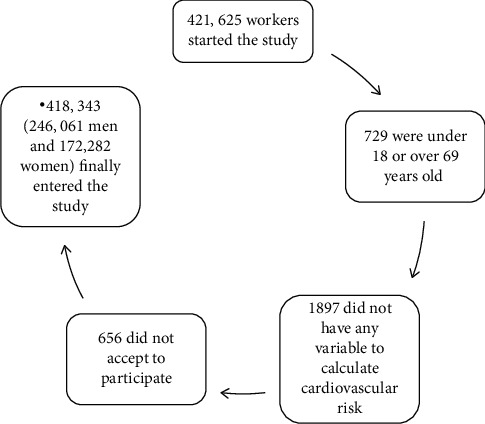
Flowchart of the participants in the study.

The present cross-sectional study included 418,343 male and female Spanish adult workers, aged between 18 and 69 years (Figure 1). The study methods have been described in detail previously [3]. Briefly, participants were recruited from periodic occupational health assessments between 2012 and 2013 in different Spanish geographic areas (Balearic Islands, Andalusia, Canary Islands, Valencia, Catalonia, Madrid, Castilla La Mancha, Castilla y León, and Basque Country). The inclusion criteria were (a) meeting the age range 18–69 years and (b) giving consent to participation in the study and permission for the use of the data for epidemiological purposes. At baseline, all subjects underwent standard health examination, anthropometric measurements, and metabolic tests.

### 2.2. Data Collection and Definition of Variables

Sociodemographic and lifestyle characteristics were collected at baseline using questionnaires. Smoking habits were also assessed, and participants were categorized as “smoker” or “nonsmoker”. Social class was defined according to the Spanish Epidemiology Society classification [13]. In general terms, Class I (upper class) includes managers and qualified professionals; Class II (middle class) includes intermediate occupations and employees; and Class III (lower class) includes manual workers.

The anthropometric measurements were recorded at baseline according to the guidelines of the International Standards for Anthropometric Assessment (ISAK) manual [14] and taken by qualified specialists or trained researchers to minimize coefficients of deviation. Body weight was measured to the nearest 0.1 kg using an electronic scale (Seca 700 scale, Hamburg); height was measured to the nearest 0.5 cm using a stadiometer (Seca 220 Telescopic Height Rod for Column Scales, Hamburg); and BMI (kg/m^2^) was calculated as weight (kg) divided by height squared (m^2^). Blood pressure was measured in triplicate, with a one-minute gap between measurements, using an electric and calibrated sphygmomanometer (OMRON M3, Healthcare Europe, Spain), with the patient in a supine position after a 10-minute rest. The mean of the three measurements was calculated and recorded.

Venous blood samples were collected at baseline from the antecubital vein after overnight fast in suitable vacutainers without anticoagulant to obtain serum. Serum concentrations of glucose, TG, gamma-glutamyl transferase (GGT), and cholesterol were measured by standard procedures using a Beckman Coulter SYNCHRON CX1 9 PRO clinical system (Brea, CA, USA).

Atherogenic dyslipidaemia was defined as triglyceride levels ≥ 150 mg/dL, HDL-c values < 40 mg/dL in men and <45 mg/dL in women, and normal LDL-c. Additionally, if LDL-c levels were >160, LT was considered [10].

### 2.3. Ethical Considerations

The study protocol was in accordance with the Declaration of Helsinki and was approved by the Ethics Committee of the Balearic Islands Health Service (CEI-IB Ref. No. 1887). Participants were informed of the purpose and requirements of the study before they provided consent to participate.

### 2.4. Methods

The scales to be used to assess overweight and obesity are the following:

- BMI, which is a mathematical ratio that associates the mass and height of an individual. It will be classified according to the SEEDO criteria: overweight from 25 kg/m^2^ and obesity from 30 kg/m^2^.
- Waist to height ratio (WtHR), establishing 0.50 [15] as the cutoff point.
- CUN-BAE. It is calculated as −44.988 + (0.503 × age) + (10.689 × gender) + (3.172 × BMI)−(0.026 × BMI2) + (0.181 × BMI × gender)−(0.02 × BMI × age)−(0.005 × BMI2 × gender) + (0.00021 × BMI2 × age), where male = 0 and female = 1 with respect to gender and age is measured in years [16].

The following classifications will be used for stratification into the different categories of overweight and obesity for male and female population: <18.5 kg/m^2^, underweight; 18.5–24.9 kg/m^2^, normal weight; 25–29.9 kg/m^2^, overweight; >30 kg/m^2^, obesity.

(i) ECORE-BF [17]: It is calculated as −97.102 + 0.123 (age) + 11.9 (gender) + 35.959 (LnBMI) where male equals 0 and female equals 1. The authors propose the same cutoff points as CUN-BAE.
(ii) Relative fat mass [18]: In women it is calculated as 76(−×20 (height/waist)) while in men it is 64(−×20 (height/waist)). Suggested cutoff points are 40% in women and 30% in men [19].
(iii) Palafolls formula [20]: It is calculated as follows: men = ([BMI/waist] × 10) + BMI; women = ([BMI/waist] × 10) + BMI + 10. The authors propose the same cutoff points as CUN-BAE.
(iv) Deurenberg fat mass index [21]. Fat mass (%) = 1.2 × BMI + 0.23 × age − 10.8 × gender − 5.4 where female equals 0 and male equals 1. Obesity is 30% or more in men and 32% or more in women.
(v) Body roundness index [22] is calculated as follows:
  (1) $$\begin{array}{c} \text{BRI}=364.2-365.5\times \sqrt{1-\frac{{\left(\text{WC}/2\pi \right)}^{2}}{{\left(0.5\text{ height}\right)}^{2}}}. \end{array}$$
(vi) Body shape index [23] is calculated as follows:
  (2) $$\begin{array}{c} \text{ABSI}=\frac{\text{WC}}{{\text{BMI}}^{\left(2/3\right)}{\text{Height}}^{\left(1/2\right)}}. \end{array}$$
(vii) Visceral adiposity index [24] is calculated as follows:
  (3) $$\begin{array}{c} \text{Females}:\text{VAI}=\left(\frac{\text{WC}}{36.58+\left(1.89\times \text{BMI}\right)}\right)\times \left(\frac{\text{TG}}{0.81}\right)\times \left(\frac{1.52}{\text{HDL}}\right),\\ \text{Males}:\text{VAI}=\left(\frac{\text{WC}}{39.68+\left(1.88\times \text{BMI}\right)}\right)\times \left(\frac{\text{TG}}{1.03}\right)\times \left(\frac{1.31}{\text{HDL}}\right). \end{array}$$

Waist circumference is expressed in cm, and LDL and triglycerides are expressed in mmol/L. The cutoff points for obesity vary with age [25].

(i) Conicity index [26] is calculated as follows:
  (4) $$\begin{array}{c} \text{Conicity index}:\frac{\text{Waist circumference}\left(\text{m}\right)}{0.109\sqrt{\text{Body weight}\left(\text{kg}\right)/\text{Height}\left(\text{m}\right)}}\\ A=\pi r^{2}. \end{array}$$
(ii) NWAI (normalized weight-adjusted index) [27]: The formula is (weight/10)−(10 × height) + 10, expressing weight in kg and height in meters.
(iii) Body surface index [28] is calculated as follows: BSI = weight/BSA
(iv) BSA (body surface area) = weight^0.425^ × height^0.725^ × 0.007184 (weight in (kg) and height in (cm)).
(v) Body fat index [29] is calculated as follows: −28.294 + 3.740_x1_ × −0.074_x2_ + 11.303_x3_ − 0.169_x4_ + 0.079_x5_ + 0.671_x6_. x1, race (1 = Asian, 2 = non-Asian); x2, age (years); x3, sex (1 = male, 2 = female); x4, height (cm); x5, weight (kg); x6, waist circumference (cm)

### 2.5. Statistical Analyses

Continuous variables with normal distribution are expressed as means (±SDs) and were compared by Student's *t*-test, whereas categorical variables are expressed as *n* (%) and 95% CI and were compared by chi-square (*χ*2) tests. The sample follows a normal distribution. The receiver operating characteristic (ROC) curve analysis was used to determine the predictive ability of overweight and obesity scales to identify AD and LT. Analyses were performed using the Statistical Package for the Social Sciences (SPSS) software version 25.0 (IBM Company, New York, NY, USA) for Windows.

## 3. Results

Baseline sociodemographic, anthropometric, and clinical characteristics of the study subjects by gender and as a whole are shown in Table 1. The sample included 418,343 individuals, comprising 246,061 men (58.8%) and 172,282 (41.2%) women, with mean age of 40.2 ± 11.0 years. The prevalence of AD in the entire sample was 6.59%. The prevalence of AD was higher among men (8.38%) than among women (4.14%). There were significant differences in anthropometrical and biochemical parameters analyzed, such as age, weight, WC, SBP, DBP, total cholesterol, LDL-c, and FPG, being significantly higher in subjects with than those without AD, in both sexes. On the contrary, HDL-c levels in individuals affected by AD are not only significantly lower than HDL-c levels in non-AD group, but also greatly decreased with respect to clinical reference values. It was noticeable that subjects without AD exhibited elevated levels in both total cholesterol and LDL-c parameters, close to the maximum clinical thresholds.

When data is stratified by age ranges, it is noticeable that the prevalence of AD rises as age increases. The greatest rate of AD is found in individuals between 50 and 70 years (11.4%). This prevalence of AD is considerably different when compared to that in the second highest prevalence group of age, which comprises individuals between 40 and 49 years old (7.4%). The youngest individuals are less prone to exhibit AD, since only 1.60% of the individuals are aged between 18 and 29 years. According to social classes, a slight increase in the prevalence of AD is observed as the social class decreases. Even though the difference in the prevalence of AD between smoking groups was statistically significant, prevalence of AD in smokers (6,41%) was comparable to that in nonsmokers (6,08%).

Mean values of overweight and obesity scales according to AD and LT values by gender are shown in Table 2.

Subjects affected by AD and LT in the study exhibited significantly higher mean values than those without AD and LT in all overweight, obesity, and body fat related scales studied (BMI, WtHR, CUN-BAE, ECORE-BF, RFM, Palafolls and Deurenberg formulae, BFI, BSI, NWAI, BRI, ABSI, VAI, and conicity index), in both genders. It is noticeable that individuals not affected by neither AD or LT exhibit mean values of BMI of 25.06 ± 4.99 kg/m^2^ and 25.24 ± 5.13 kg/m^2^, respectively, in women and 26.28 ± 4.21 kg/m^2^ and 26.57 ± 4.41 kg/m^2^, respectively, in men, which would classify them as overweight according to BMI scale. Nonetheless, when BMI mean values are assessed in AD or LT people, both sexes exhibit values higher than 30 kg/m^2^, which would classify them as obese.

Percentages of prevalence of elevated values of obesity scales, according to AD and LT, are shown in Table 3, segregated by gender.

It is noticeable that a great percentage of people affected by AD and LT are classified as obese according to different obesity scales, such as WtHR > 0.5, BMI, CUN-BAE, ECORE-BF, RFM, Palafolls, and Deurenberg. In other words, when AD is present, the prevalence of higher values in obesity scales is observed. Then, the probability of being classified as obese according to obesity scales when AD and LT are present is higher compared to those subjects not affected by these two forms of lipid disbalance. The wide difference between common scales such as BMI and other scales such as Palafolls is noteworthy. Concretely, the percentage of obese women according to BMI is 20.05% and 21.23% in absence of AD and LT, respectively, while it increases up to 57.47% and 52.62% in women with AD or LT, respectively, according to Palafolls formula. Similar tendency is detected in men: 38.47% and 40.72% of the individuals without AD or LT, respectively, are classified as obese using the BMI scale, while 77.60% and 76.98% of those affected by AD or LT, respectively, are obese according to BMI. Nonetheless, when obesity is assessed following Palafolls formula, more cases of obesity are targeted. Concretely,95.64% women with AD and 95.51% of women with LT are classified as obese according to Palafolls formula. Furthermore, 98.41% and 72.69% of the women without AD are also classified as obese according to Palafolls formula. Similar results can be spotted in men when using Palafolls parameters to diagnose obesity: 98.41% and 98.61% of men with AD or LT, respectively, would exhibit obesity, while those without AD or LT would be 86.02% and 86.71%, respectively.

**Figure 2:**
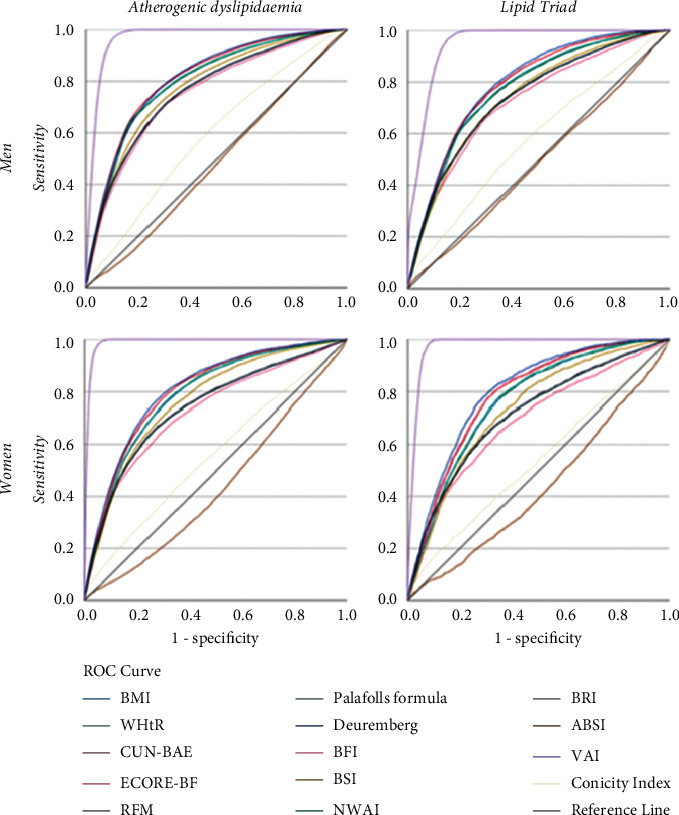
Receiver operating characteristic (ROC) curves for the prognostic value of different variables in predicting AD and LT.

Receiver operating characteristic (ROC) curves for the prognostic value of different variables in predicting AD and LT are shown in Figure 2. Area under the ROC curve (AUC) according to different overweight and obesity scales is shown in Table 4.

Our ROC analysis of participants (Figure 2, Table 4) who fulfilled the criteria for the two studied forms of dyslipidaemias indicated that VAI was the strongest predictor of AD (AUC = 0.934, 95% CI: 0.933 to 0.936) and LT (AUC = 0.926, 95% CI: 0.923 to 0.928) in both men and women. On the contrary, ROC analysis showed that BMI and NWAI had a lower value than VAI for prediction of progression of AD (AUC = 0.800, 95% CI: 0.797 to 0.803 for BMI; AUC = 0.800, 95% CI: 0.798 to 0.803 for NWAI) and LT (AUC = 0.775, 95% CI: 0.770 to 0.780 for both BMI and LT). The rest of the parameters assessed showed limited prediction capacity for AD and LT, with their AUC being lower than 0.75. The ABSI exhibited the lowest predictive value (AUC = 0.509, 95% CI: 0.506 to 0.513).

Tables 5 and 6 show cutoff points, sensibility, specificity, and Youden index of different overweight and obesity scales in women and men, respectively, affected by AD and LT. It is noticeable that cutoff value for BMI to define AD and LT in women is 27.1, in both cases.

## 4. Discussion

It is generally accepted that obesity entails alterations in lipid biochemistry, progressing toward dyslipidaemia atherogenesis, a crucial feature in the development of cardiovascular events [10]. The present work was established to evaluate the linkage of obesity scales with two forms of lipidic metabolic disbalances, as a tool for the early detection of obesity and associated lipid disorders. Concretely, this large cohort study assessed the relationship of two forms of dyslipidaemia, namely, AD and LT, with different scales of overweight and obesity in 418,343 Spanish workers.

AD is of great interest as it is associated with different diseases that are currently widely prevalent in the general population and are accompanied by a high cardiovascular risk, such as overweight (37%), obesity (17%), diabetes (14%), and metabolic syndrome (30%) [30]. Prevalence of AD in our sample, comprising 418,343 Spanish adult workers, was 6.2%. According to epidemiological available data, this result is relatively low when compared to high-risk cohorts, since AD in Spanish population is present in 34% of diabetics, 10% of hypertensive individuals, and 21–34% of patients with a history of vascular disease (coronary, cerebral, or peripheral arterial) [11]. This can be explained by the features of our sample, as it comprises general working population with no specific pathology in common. Furthermore, we spotted 16.36% of obese women and 19.61% of obese men according to BMI scale, which is the most common diagnostic tool that healthcare providers use to identify obesity. Our data in Spanish workers is in accordance with current records, since the association between dyslipidaemia, obesity, and hypertension is well established [2, 31], and all have been described to be risk factors for cardiovascular disease (CVD) [32]. The dyslipidaemia associated with obesity plays a major role in the development of atherosclerosis and CVD in obese individuals [33]. Indeed, all the components of the dyslipidaemia, including higher TG, decreased HDL-c levels, and increased small, dense LDL-c particles, have been shown to be atherogenic [34]. Of note, the major cardiovascular-protective role for HDL-c has been attributed to reverse cholesterol transport, which carries excess cholesterol in arterial macrophages to the liver for excretion. Thus, decreased level of HDL-c can lead to disbalances of cardiovascular homeostasis [35]. Weight loss and regular physical activity, even if they do not result in a body weight loss, can potentially improve this dyslipidaemic status and thus lower the CVD risk [36]. In addition, overweight and, specially, obese individuals should be targeted for lipid-lowering interventions, when required. Then, workplace emerges as a suitable setting for early detection of individuals at risk of developing harmful lipid abnormalities associated with overweight and obesity such as AD and LT. Labor health assessments should aim to target workers at risk, identify subjects who can benefit most from lifestyle changes, and promote healthy behaviors that can ameliorate the development of AD and LT in general population.

We spotted a cutoff value of 27.1 for BMI to define AD and LT in women. This would mean that individuals with moderate-to-high overweight, according to BMI scale, are at increased risk of suffering from AD and/or LT. This statement is concordant with prior findings that conclude that lipid abnormalities are typical features of the metabolic syndrome [37] and may be associated with proinflammatory cytokine released by the adipose tissue that could affect the endothelium dynamics and promote atherogenic processes. [2]. Furthermore, the development of insulin resistance in peripheral tissues seems to have a major association with obesity, metabolic syndrome (MetS), and dyslipidaemia [38].

CUN-BAE is considered the gold standard method to estimate body fat percentage in the general population [5]. Obesity is defined when CUN-BAE values are above 35% in women and 25% in men. We obtained values under obesity thresholds in men (24.9%) and women (34.86%) not affected by AD. On the contrary, CUN-BAE values were beyond threshold in men (32.25%) and women (42.86%) with AD. Concretely, we identified cutoff values for CUN-BAE for defining AD and LT. Cutoff value, for both AD and LT, was 28.8 in women and 39.1 in men. These results provide evidence that adiposity, according to the CUN-BAE formula, and biochemical analysis of predictive factors of obesity and lipidic disbalances together represent useful tools in assessing the risk of cardiovascular disease.

Amato introduced visceral adiposity index (VAI), which is a formula that includes WC, BMI, TG, and HDL-c [24, 39]. VAI is a consistent index for the function of visceral fat. Prior researches have validated the accuracy of VAI for predicting various noncommunicable diseases including MetS, type 2 diabetes (T2D), and hypertension [40, 41]. Preceding studies conducted in Caucasian Sicilian population established a VAI cutoff value of 2 associated with cardiometabolic risk [42], and another study defined a VAI cutoff of 1.9 for describing dysfunctional adiposity in Venezuelan population [43]. We obtained cutoff values for defining AD and LT. A cutoff of 4.8 was spotted in both AD and LT in women, while in men the cutoff value was 11.8 for AD and 12.3 for LT. VAI has been proven to be an indicator of adipose distribution and a function that indirectly expresses cardiometabolic risk [25]. According to literature, VAI values higher than 3 are considered to be indicators of severe adipose tissue disfunction. Thus, AD would be associated with harmful complications related to adipose tissue according to obtained cutoff values. Furthermore, VAI presents an association with MetS components in males and females with an increased risk of abdominal obesity, hypertriglyceridemia, and low HDL-c, proving to be a good MetS component predictor even among healthy young adults [44]. Our work reinforces the idea that VAI is a good predictor of metabolic disbalances in general population, as our sample includes individuals from 18 to 69 years. The main limitation of the study is that it was conducted in working population (aged 20–69 years), so the results cannot be extrapolated to the general population.

In conclusion, to the best of our knowledge, no studies have been conducted on the association of AD and LT to scales of overweight and obesity. Our work indicates that AD and LT positively correlate with different scales of overweight and obesity. Further studies should aim to identify other contributory factors. Our obtained data might be useful in laying the groundwork for future works on atherogenic dyslipidaemia and lipid triad.

## Figures and Tables

**Table 1:** Sociodemographic, anthropometric, clinical, and analytical characteristics of the people by gender.

	Women			Men			Total		
	No AD	AD	*p*	No AD	AD	*p*	No AD	AD	*p*
	*n* = 165,431	*n* = 6,851		*n* = 227,030	*n* = 19,031		*n* = 392,461	*n* = 25,882	
	Mean (SD)	Mean (SD)		Mean (SD)	Mean (SD)		Mean (SD)	Mean (SD)	

Age (years)	39.30 (10.72)	46.33 (9.99)	<0.0001	40.07 (11.05)	46.51 (9.34)	<0.0001	39.75 (10.92)	46.46 (9.52)	<0.0001
Height (cm)	161.88 (6.47)	160.48 (6.66)	<0.0001	174.67 (6.95)	173.96 (7.10)	<0.0001	169.28 (9.25)	170.39 (9.18)	<0.0001
Weight (kg)	65.68 (13.58)	79.55 (16.54)	<0.0001	80.23 (13.97)	94.86 (16.50)	<0.0001	74.10 (15.57)	90.91 (17.84)	<0.0001
WC (cm)	74.40 (10.26)	83.85 (13.19)	<0.0001	85.39 (10.71)	95.37 (11.39)	<0.0001	80.76 (11.84)	92.32 (12.94)	<0.0001
SBP 8 (mm Hg)	117.00 (15.41)	127.67 (17.89)	<0.0001	127.50 (15.22)	136.19 (16.85)	<0.0001	123.07 (16.16)	133.93 (17.53)	<0.0001
DBP (mm Hg)	72.33 (10.29)	78.82 (11.06)	<0.0001	77.23 (10.79)	83.95 (11.00)	<0.0001	75.16 (10.86)	82.59 (11.25)	<0.0001
Cholesterol (mg/dL)	189.36 (35.13)	219,33 (38.75)	<0.0001	190.64 (38.07)	215.31 (41.04)	<0.0001	190.10 (36.87)	216.79 (40.52)	<0.0001
HDL (mg/dL)	57.18 (8.53)	46.37 (3.76)	<0.0001	51.41 (7.85)	37.03 (2.55)	<0.0001	53.84 (8.63)	39.50 (5.05)	<0.0001
LDL (mg/dL)	115.37 (34.46)	134.32 (38.06)	<0.0001	116.83 (36.17)	133.24 (39.74)	<0.0001	116.21 (35.46)	133.53 (39.29)	<0.0001
TG (mg/dL)	84.22 (36.95)	207.09 (78.57)	<0.0001	113.98 (75.50)	239.83 (117.83)	<0.0001	101.44 (63.95)	231.17 (109.78)	<0.0001
FPG (mg/dL)	87.32 (14.07)	99.22 (29.18)	<0.0001	92.31 (19.60)	104.88 (33.46)	<0.0001	90.21 (17.65)	103.38 (32.48)	<0.0001
GGT (U/L)	19.91 (18.60)	32.99 (35.71)	<0.0001	34.22 (36.92)	54.43 (57.56)	<0.0001	28.29 (31.49)	49.00 (53.70)	<0.0001

	(%)	(%)	*p*	(%)	(%)	*p*	(%)	(%)	*p*
18–29 years	97,98	2,02	<0.0001	98,54	1,46	<0.0001	98.40	1.60	<0.0001
30–39 years	96,15	3,85		95,93	4,07		96.03	3.97	
40–49 years	93,21	6,79		92,36	7,64		92.59	7.41	
50–69 years	87,21	12,79		89,31	10,69		88.57	11.43	
Class I	96,33	3,67	<0.0001	94,31	5,69	0.033	95.27	4.73	<0.0001
Class II	94,33	5,67		93,78	6,22		94.36	5.64	
Class III	93,39	6,61		93,79	6,21		93.56	6.44	
Nonsmokers	93,78	6,22	0.235	93,97	6,03	<0.0001	93.92	9.08	<0.0001
Smokers	93,89	6,11		93,51	6,49		93.59	9.41	

Results are reported as mean ± SD or *n* (%). WC: waist circumference; SBP: systolic blood pressure; DBP: diastolic blood pressure; TG: triglycerides; FPG: fasting plasma glucose. Classes I (upper), II (middle), and III (lower) refer to social classes defined according to the Spanish Epidemiology Society classification [13].

**Table 2:** Mean values of overweight and obesity scales according to AD and LT values by gender.

	Women				Men			
	No AD	AD	No LT	LT	No AD	AD	No LT	LT
	*n* = 165,431	*n* = 6,851	*n* = 170,566	*n* = 1,716	*n* = 227,030	*n* = 19,031	*n* = 240,669	*n* = 5,392
	Mean (SD)	Mean (SD)	Mean (SD)	Mean (SD)	Mean (SD)	Mean (SD)	Mean (SD)	Mean (SD)
BMI (kg/m^2^)	25.06 (4.99)	30.85 (5.94)	25.24 (5.13)	30.16 (5.54)	26.28 (4.21)	31.30 (4.82)	26.57 (4.41)	31.06 (4.84)
WtHR	0.46 (0.06)	0.52 (0.08)	0.46 (0.06)	0.52 (0.08)	0.49 (0.06)	0.55 (0.06)	0.49 (0.06)	0.55 (0.06)
CUN-BAE	34.86 (6.98)	42.86 (6.42)	35.10 (7.11)	42.42 (6.00)	24.90 (6.36)	32.28 (5.81)	25.33 (6.56)	32.06 (5.74)
ECORE-BF	34.82 (7.09)	43.16 (6.94)	35.08 (7.23)	42.72 (6.58)	24.93 (6.04)	32.03 (5.61)	25.34 (6.25)	31.84 (5.55)
RFM	31.78 (5.44)	36.84 (5.94)	31.93 (5.53)	36.36 (5.80)	22.52 (4.88)	27.05 (4.25)	22.77 (4.96)	26.96 (4.34)
Palafolls formula	38.43 (5.29)	44.54 (6.18)	38.62 (5.43)	43.84 (5.77)	29.36 (4.42)	34.58 (5.04)	29.66 (4.62)	34.34 (5.07)
Deurenberg formula	33.71 (6.88)	42.28 (7.45)	33.97 (7.06)	42.03 (7.00)	24.55 (6.17)	32.06 (6.21)	24.98 (6.42)	31.93 (6.09)
BFI	26.63 (7.59)	33.79 (9.82)	26.86 (7.78)	32.49 (9.24)	21.64 (7.74)	29.14 (8.43)	22.07 (7.98)	28.75 (8.50)
BSI	50.19 (7.86)	58.53 (9.23)	50.45 (8.06)	57.15 (8.48)	57.21 (7.48)	65.36 (8.50)	57.68 (7.79)	64.72 (8.52)
NWAI	0.38 (1.33)	1.91 (1.54)	0.43 (1.36)	1.73 (1.41)	0.56 (1.29)	2.09 (1.47)	0.64 (1.35)	2.02 (1.46)
BRI	2.71 (1.15)	3.91 (1.61)	2.74 (1.19)	3.77 (1.53)	3.22 (1.11)	4.38 (1.30)	3.28 (1.15)	4.36 (1.32)
ABSI	0.069 (0.06)	0.068 (0.06)	0.069 (0.01)	0.068 (0.01)	0.074 (0.06)	0.073 (0.06)	0.074 (0.01)	0.073 (0.01)
VAI	2.51 (1.18)	7.50 (3.27)	2.65 (1.14)	8.41 (5.23)	6.34 (4.64)	20.01 (10.68)	7.01 (5.47)	24.44 (16.80)
Conicity index	1.08 (0.09)	1.09 (0.10)	1.08 (0.09)	1.09 (0.10)	1.16 (0.09)	1.19 (0.09)	1.16 (0.09)	1.19 (0.10)

*p*-value in all cases <0.0001. AD: atherogenic dyslipidaemia; LT: lipid triad; BMI: body mass index; WtHR: waist to height ratio; CUN-BAE: University of Navarra Clinic-Body Fat Estimator; ECORE-BF; Equation Córdoba for Estimation of Body Fat, RFM: relative fat mass; BFI: body fat index; BSI: body surface index; NWAI: normalized weight-adjusted index; BRI: body roundness index; ABSI: body shape index; VAI: visceral adiposity index.

**Table 3:** Prevalence of elevated values of obesity scales according to AD and LT values by gender.

	No AD	AD	No LT	LT	No AD	AD	No LT	LT
	Women				Men			
	*n* = 165,431	*n* = 6,851	*n* = 170,566	*n* = 1,716	*n* = 227,030	*n* = 19,031	*n* = 240,669	*n* = 5,392
	(%)	(%)	(%)	(%)	(%)	(%)	(%)	(%)
WtHR > 0.50	20.05 (20.00–20.11)	57.47 (55.37–59.61)	21.23 (21.17–21.29)	52.62 (49.58–55.66)	38.47 (38.44–38.50)	77.60 (76.18–79.11)	40.72 (40.70–40.75)	76.08 (73.89–78.30)
BMI obesity	14.80 (14.75–14.82)	53.93 (51.80–56.04)	16.04 (15.99–16.10)	47.55 (44.50–50.61)	15.96 (15.93–16.00)	63.09 (65.58–64.63)	18.71 (18.69–18.73)	59.63 (57.44–61.85)
CUN-BAE obesity	46.17 (46.11–46.21)	88.35 (86.20–90.49)	47.44 (47.38–47.50)	88.11 (85.06–91.18)	48.72 (48.69–48.75)	89.26 (87.74–90.82)	51.02 (51.0–51.05)	89.13 (86.87–91.34)
ECORE-BF obesity	45.18 (45.12–45.24)	87.61 (85.50–89.84)	46.46 (46.40–46.52)	87.18 (84.15–90.23)	48.65 (48.62–48.68)	89.26 (87.73–90.81)	50.95 (50.93–50.97)	89.11 (86.84–91.32)
RFM obesity	32.38 (32.33–32.43)	70.46 (68.33–72.60)	33.56 (33.51–33.62)	67.07 (64.00–70.11)	49.08 (49.05–49.11)	83.64 (82.11–85.12)	51.06 (51.04–51.08)	82.51 (80.30–84.74)
Palafolls obesity	98.41 (98.34–98.48)	95.64 (93.50–97.87)	72.69 (72.62–72.76)	95.51 (92.47–98.54)	86.02 (86.00–86.05)	98.41 (96.90–99.93)	86.71 (86.69–86.74)	98.61 (96.40–100.00)
Deurenberg obesity	67.36 (67.30–67.42)	95.85 (93.60–90.03)	68.21 (68.14–68.28)	96.85 (93.77–99.91)	44.88 (44.85–44.91)	88.23 (86.70–89.75)	47.32 (47.30–47.34).	88.67 (86.47–90.81)

*p*-value in all cases <0.0001. AD: atherogenic dyslipidaemia; LT: lipid triad; BMI: body mass index; WtHR: waist to height ratio; CUN-BAE: University of Navarra Clinic-Body Fat Estimator; ECORE-BF: Equation Córdoba for Estimation of Body Fat; BMI: body mass index; RFM: relative fat mass.

**Table 4:** Area under the ROC curve according to different overweight and obesity scales in the whole sample.

	Atherogenic dyslipidaemia		Lipid triad	
	ROC area	95% CI	ROC area	95% CI
BMI	0.800	0.797–0.803	0.775	0.770–0.780
WtHR	0.767	0.764–0.770	0.747	0.741–0.753
CUN-BAE	0.707	0.705–0.710	0.684	0.679–0.689
ECORE-BF	0.706	0.703–0.709	0.683	0.678–0.689
RFM	0.634	0.631–0.637	0.614	0.608–0.620
Palafolls formula	0.668	0.665–0.671	0.641	0.635–0.647
Deurenberg formula	0.721	0.718–0.724	0.702	0.696–0.707
BFI	0.718	0.715–0.722	0.688	0.682–0.695
BSI	0.781	0.778–0.783	0.752	0.747–0.757
NWAI	0.800	0.798–0.803	0.775	0.770–0.780
BRI	0.767	0.764–0.770	0.747	0.742–0.753
ABSI	0.509	0.506–0.513	0.515	0.508–0.522
VAI	0.934	0.933–0.936	0.926	0.923–0.928
Conicity index	0.613	0.610–0.617	0.607	0.600–0.613

Area under the ROC curve according to different overweight and obesity. AD: atherogenic dyslipidaemia; LT: lipid triad; BMI: body mass index; WtHR: waist to height ratio; ECORE-BF : Equation Córdoba for Estimation of Body Fat; RFM: relative fat mass; BFI: body fat index; BSI: body surface index; NWAI: normalized weight-adjusted index; BRI: body roundness index; ABSI: body shape index; VAI: visceral adiposity index.

**Table 5:** Cutoff, sensibility, specificity, and Youden index of different overweight and obesity scales in men.

	Men				*n* = 246.061			
	Atherogenic dyslipidaemia				Lipid triad			
	Cutoff	Sensibility	Specificity	Youden index	Cutoff	Sensibility	Specificity	Youden index
BMI	28.3	74.2	73.2	0.474	28.9	72.1	72.0	0.410
WtHR	0.52	71.2	69.7	0.409	0.52	69.6	67.3	0.369
CUN-BAE	28.8	74.7	74.6	0.493	28.8	72.1	71.9	0.400
ECORE-BF	28.7	75.1	74.5	0.496	28.8	72.4	72.4	0.480
RFM	25.3	70.8	70.2	0.410	25.3	68.7	68.3	0.370
Palafolls formula	31.5	73.6	73.6	0.472	31.5	71.0	71.0	0.420
Deurenberg formula	28.2	74.5	74.5	0.490	28.4	72.7	72.6	0.430
BFI	25.1	70.4	70.4	0.408	25.1	68.0	67.7	0.350
BSI	60.5	70.8	70.6	0.414	60.5	68.7	68.6	0.373
NWAI	1.20	73.5	72.9	0.464	1.20	70.8	70.2	0.410
BRI	3.7	70.4	70.3	0.407	3.7	68.4	68.4	0.368
ABSI	0.073	49.9	48.4	−0.170	0.073	49.8	49.5	−0.070
VAI	11.8	92.3	92.0	0.843	12.3	89.3	88.3	0.776
Conicity index	1.17	56.9	56.8	0.137	1.17	56.4	56.0	0.124

AD: atherogenic dyslipidaemia; LT: lipid triad; BMI: body mass index; WtHR: waist to height ratio; ECORE-BF: Equation Córdoba for Estimation of Body Fat; RFM: relative fat mass; BFI: body fat index; BSI: body surface index; NWAI: normalized weight-adjusted index; BRI: body roundness index; ABSI: body shape index; VAI: visceral adiposity index.

**Table 6:** Cutoff, sensibility, specificity, and Youden index of different overweight and obesity scales in women.

	Women				*n* = 172,282			
	Atherogenic dyslipidaemia				Lipid triad			
	Cutoff	Sensibility	Specificity	Youden index	Cutoff	Sensibility	Specificity	Youden index
BMI	27,1	72,5	72,5	0,450	27,1	71,1	69,8	0,409
WtHR	0,48	68,2	67,4	0,356	0,48	67,2	66,2	0,334
CUN-BAE	39,1	73,8	73,7	0,475	39,1	72,9	72,4	0,453
ECORE-BF	39,1	73,9	73,8	0,477	39,1	72,7	72,7	0,454
RFM	34,1	69,4	69,0	0,384	34,1	67,1	66,4	0,335
Palafolls formula	40,7	72,9	72,3	0,452	40,7	70,4	70,4	0,408
Deurenberg formula	37.6	74,7	74,6	0,493	37.6	74,1	74,1	0,482
BFI	28.3	67,7	67,5	0,352	28.3	64,8	64,7	0,295
BSI	53.1	70,6	70,5	0,411	53.1	68,5	68,4	0,369
NWAI	0.95	72,6	72,5	0,451	0.95	69,8	69,6	0,394
BRI	3.0	69,3	69,2	0,385	3.0	66,8	66,6	0,334
ABSI	0.068	45,1	45,1	−0,098	0.068	44,5	44,4	−0,111
VAI	4.8	96,0	95,9	0,919	4.8	95,7	93,1	0,888
Conicity index	1.08	54,0	53,9	0,079	1.08	52,0	51,9	0,039

AD: atherogenic dyslipidaemia; LT: lipid triad; BMI: body mass index; WtHR: waist to height ratio; RFM: relative fat mass; BFI: body fat index; BSI: body surface index; NWAI: normalized weight-adjusted index; BRI: body roundness index; ABSI: body shape index; VAI: visceral adiposity index.

## Data Availability

The data used to support the findings of this study have not been made available because the authors did not agree with the data being shared publicly.
